# Treatment of Budd-Chiari syndrome with inferior vena cava thrombosis

**DOI:** 10.3892/etm.2013.961

**Published:** 2013-02-18

**Authors:** RUIHUA WANG, QINGYI MENG, LIFENG QU, XUEJUN WU, NIANFENG SUN, XING JIN

**Affiliations:** 1 Department of Vascular Surgery, Jinan Central Hospital, Shandong University, Jinan 250021, P.R. China; 2 Department of Vascular Surgery, Shandong Provincial Hospital, Shandong University, Jinan 250021, P.R. China

**Keywords:** Budd-Chiari syndrome, inferior vena cava, thrombosis, classification

## Abstract

The aim of this study was to evaluate the initial results of 41 patients with Budd-Chiari syndrome (BCS) with inferior vena cava (IVC) thrombosis, with regard to the clinical safety and feasibility of the therapeutic approaches selected according to the classification of the condition. Forty-one patients with BCS and IVC thrombosis were admitted for retrospective analysis. All 41 patients were classified as having one of three types of BCS. Interventional therapy was used successfully in 28 patients (68.3%), 7 patients (17.1%) were given conservative treatment and 6 patients (14.6%) were treated with surgical shunts. The interventional approach was used in 29 patients in total and was successful in 28 patients (all those of types I and II, and 3 of the 4 patients of type III with acute thrombosis; 96.6%). None of these 28 patients had pulmonary embolism, pericardial tamponade or intra-abdominal bleeding. After 1–5 years, 4 patients (9.8%) had a second dilation of the IVC. In the 7 cases treated in a conservative manner, 2 cases succumbed to upper gastrointestinal bleeding and 1 case succumbed to liver and kidney failure. This study indicates that the classification of BCS patients with IVC thrombosis is helpful in selecting a therapeutic approach. Interventional therapy is the first therapeutic choice for BCS patients with IVC thrombosis of type I, type II or type III with acute thrombosis. For the patients of type III with an obsolete thrombus, surgical shunts or conservative treatment are the main therapeutic methods.

## Introduction

Budd-Chiari syndrome (BCS) results from venous obstruction (occlusion or stenosis) of the hepatic veins and/or retrohepatic inferior vena cava (IVC) and presents clinically as portal and IVC hypertension (1). There have been few reports on the subject from the United States and Europe, but there have been several reports from developing countries such as China and India. It is common in China’s lower reaches of the Yellow River such as the Shandong, Jiangsu and Anhui Provinces (1,2). In the West, most cases result from thrombosis of the small centrilobular or main hepatic veins (3,4). In China, however, membranous obstruction of the IVC or the hepatic vein, or both, is the most common cause of BCS and accounts for up to 60–70% of the total patient number (5–7). Thromboses are easily formed in these patients due to obstruction at the suprahepatic IVC, slow and reversed blood flow in the IVC, and the hypercoagulable blood state.

Due to the increase in the understanding and diagnosis of BCS, a greater number of BCS cases are treated, while the treatment methods remain varied. Among them, interventional therapy has been rapidly developed due to being minimally invasive (8,9). Percutaneous transluminal angioplasty, including balloon angioplasty and stent placement, has recently been recommended as a first-line treatment for obstructed IVC at or above the hepatic level in primary BCS (6,10). Patients with IVC thrombosis, particularly patients with new thromboses, remain difficult to treat, however. However, a long time ago, there was a contraindication of interventional therapy. There are many therapeutic approaches to treating IVC thrombosis. These include anticoagulation with warfarin (11), thrombolytic agents (such as the acylated streptokinase-plasminogen complex, urokinase, streptokinase and tissue plasminogen activator) administered either through a peripheral intravenous or catheter-mediated route (12–14), balloon angioplasty and/or stents (13,14) or transjugular intrahepatic portosystemic shunts (15). Surgical shunts (16) and orthotopic liver transplantation (17) have also been successfully applied for the treatment of BCS with thrombosis. They are complicated to use, however, with a high risk of causing bleeding, and a significant risk of pulmonary embolism. There has been no guidance for the selection of these methods for use in patients with BCS combined with IVC thrombosis.

**Figure 1:**
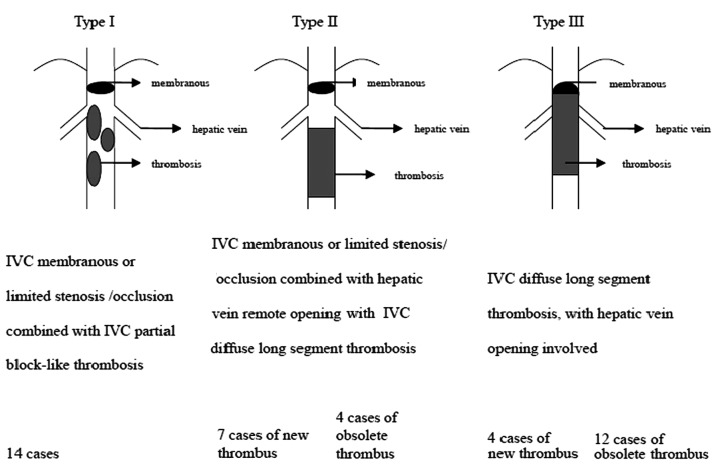
Forty-one patients with Budd-Chiari syndrome (BCS) and inferior vena cava (IVC) thrombosis were divided into three types.

Since 2006, at the vascular surgery centers in Jinan Central Hospital and Shandong Provincial Hospital, China, a set of treatment procedures for the interventional therapy of patients with IVC thrombosis has been implemented. In order to facilitate clinical standard treatment, the patients with BCS and IVC thrombosis were divided into three types (Fig. 1), and different treatments were employed. The clinical data from 41 patients with BCS and IVC thrombosis were evaluated. The purpose of this study was to evaluate the initial results with regard to the clinical safety and feasibility of the therapeutic approaches, selected according to our classification of the condition.

## Materials and methods

### Clinical data

The inclusion criteria were as follows. From November, 2006 to November, 2010, 41 cases of BCS with IVC thrombosis underwent vascular surgery at Shandong Provincial Hospital, Shandong University and Jinan Central Hospital, Shandong University. All participants were examined initially by color ultrasonography, enhanced CT, MRI or other imaging methods and diagnosed to have BCS with IVC thrombosis. The patients had an average age of 47 years, with clinically refractory ascites, hepatosplenomegaly, varicose veins of the abdominal wall and the lower extremities, and leg pigmentation or ulceration. Those diagnosed as having liver neoplasm or severe heart failure, who had long segment occlusion of the hepatic vein or whose scope of the IVC thrombosis involved the iliac and femoral vein were excluded. The patients were classified based on imaging examination results (Fig. 1). This study was conducted in accordance with the Declaration of Helsinki and with approval from the Ethics Committee of Shandong Provincial Hospital and Jinan Central Hospital. Written informed consent was obtained from all participants.

### Treatment method

Upon admission, all patients routinely underwent puncture of the right femoral vein and/or right jugular vein for orthophoria angiography and lateral radiography. This was to evaluate the scope of IVC occlusion and show the nature of the thrombus, the extent of hepatic vein patency, collateral circulation and other aspects of the disease.

**Figure 2:**
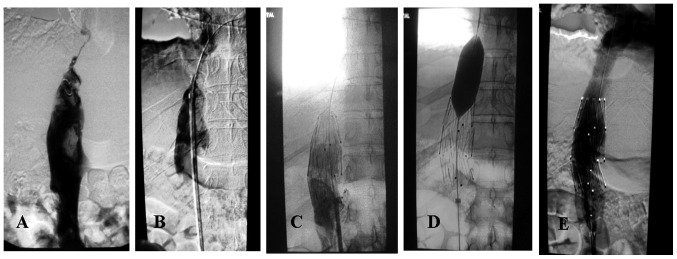
Type I inferior vena cava (IVC)-limited stenosis combined with IVC partial block-like thrombosis. (A) There was residual mural thrombus on the IVC wall. A stent was implanted for mechanical compression (B–C), and then changed to a balloon catheter with a larger caliber (25 mm) to fully expand the blocked section (D–E).

In patients of type I, the direction of membrane rupturing was selected based on the section shape of the remote and proximal ends of the IVC obstruction. Membrane rupturing was successfully coordinated with a guide wire and catheter together (8 cases were from bottom to top and 6 cases were from top to bottom). A balloon with a diameter of 5–7 mm was inserted through the blocked section to make a small hole, and a thrombolytic catheter (5 F) was put into the residual thrombus. Urokinase (100,000 U/q8h) was injected using a pulse tube, and sodium heparin solution (15 U/kg.h) was continuously injected after urokinase was injected, during which time coagulation, routine blood tests and liver and renal function were rechecked. Depending on the results, the dose was adjusted to limit the activated partial thromboplastin time to twice the normal value. Every 48 h, the radiography was checked to observe the thrombolytic effect; the total observation time did not exceed 10 days. The phlebography was checked to ensure there were no free thrombi and the IVC thrombolytic catheter was then removed. If there was residual mural thrombosis on the IVC wall, a stent was implanted for mechanical compression and changed to a balloon catheter with a larger caliber (20–30 mm) to fully expand the blocked section (Fig. 2). Patients whose stenosis remained >30% after many expansions, whose IVC pressure gradient reduced by <40% or who had a floating intimal flap, were considered for vena cava stent implantation. After surgery, anticoagulant treatment with oral warfarin was routinely administered for 3–6 months.

**Figure 3:**
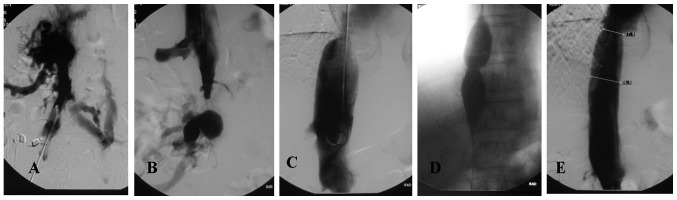
Type II inferior vena cava (IVC)-limited stenosis combined with hepatic vein remote openings and IVC diffuse long segment thrombosis. There is evident collateral circulation (A–C). A balloon catheter with a large caliber (25 mm) was used to fully expand the stenotic section (D and E) via the IVC stenosis section at the end proximal to the hepatic vein opening from top to bottom.

In the patients of type II who were revealed to have an obsolete thrombus by radiography, the stenotic section of the IVC at the end proximal to the hepatic vein was accessed from top to bottom and a balloon catheter with a large caliber (20–30 mm) was used to fully expand the stenotic section. The long segment of IVC thrombosis at the remote end of the hepatic vein opening was not processed (Fig. 3). If the condition was combined with a lower extremity refractory ulcer, a cavoatrial shunt was performed. In the patients who were demonstrated by radiography to have a new thrombus, a catheter of large caliber (12–16 F) was inserted into the thrombus via the right femoral vein. Manual mechanical suction was performed to remove most of the thrombus. Treatment was administered with a thrombolytic catheter, in a similar manner to that carried out in patients of type I before the 10 day cut-off. If there was a residual mural thrombus on the IVC wall, a stent was implanted for mechanical compression and changed to a balloon catheter with a larger caliber (20–30 mm) to fully expand the blocked section. Anticoagulant treatment with oral warfarin was administered postoperatively for 6–12 months.

For the patients of type III who were revealed to have a long segment of obsolete thrombosis by radiography and in whom the thrombosis was difficult to detect using the guide wire, a mesocaval shunt or cavoatrial shunt was used instead of endovascular treatment. The type III patients who were identified as having a newly developed thrombosis were treated as the patients of type II. In patients with stenosis or occlusion in the short segment of the hepatic vein opening, a balloon was applied to expand the lesion and a stent was implanted. If it was difficult to enter the hepatic vein from the vena cava, percutaneous hepatic vein puncture was adopted.

## Results

The 14 cases of type I were successfully treated. One case was implanted with a stent due to a visible floating intimal flap in the lumen following dilation. Five cases that had residual mural thrombi in the IVC following thrombolysis were stent implanted for mechanical compression.

The 7 cases of type II with acute thrombosis of the IVC were successfully treated following thrombolysis, among which 3 cases had IVC residual mural thrombus, and an inner stent was pre-implanted for mechanical compression; in the 4 cases of type II with obsolete thrombosis, the stenotic segment of the IVC at the end proximal to the hepatic vein was catheterized.

Of the 4 cases of type III with acute thrombosis, 3 cases were successfully treated by thrombolysis (in combination with percutaneous transhepatic puncture in 2 cases) and one case was changed to conservative treatment due to the effect of thrombolysis. In the 12 cases with obsolete thrombosis, 2 cases had an occluded hepatic vein but unobstructed accessory hepatic vein and were treated by chamber atrial shunt, while 4 cases with an occluded hepatic vein and accessory hepatic vein but unobstructed remote end of the IVC were treated by mesocaval shunt, and 6 cases were received conservative treatment as a result of poor health.

No pulmonary embolism, pericardial tamponade or intra-abdominal bleeding occurred in the successfully treated patients. All patients of type I and those of types II and III with acute thrombosis, whose symptoms and signs showed significant improvement following the successful surgery, demonstrated a reduction of ascites and the easing of lower limb and abdominal wall superficial veins. Following treatment of the IVC stenosis at the end proximal to the hepatic vein, ascites notably subsided in the type II patients with obsolete thrombosis; one case with intractable ulcers of both lower extremities was given a cavoatrial shunt, with the ulcer healing one month after surgery. During the perioperative period there were no mortalities among those who had a mesocaval or cavoatrial shunt.

Among the 41 cases studied, 38 cases were followed up for 1–5 years, with an average follow-up of 2.5 years. Three cases of type I had IVC restenosis and a secondary balloon dilation. One case of type II had IVC stent restenosis and was treated by secondary balloon dilation and one case treated with intestinal bypass surgery was changed to conservative treatment due to occlusion of the vascular bypass. In the 7 cases treated in a conservative manner, 2 cases succumbed to upper gastrointestinal bleeding and 1 case succumbed to liver and kidney failure.

## Discussion

Obstruction of the hepatic vein by thrombosis in BCS is prevalent in the United States and Europe. The majority of cases have clear underlying causes relating to oral contraception, pregnancy, polycythemia, abnormal bone marrow histiocytosis, antiphospholipid syndrome, paroxysmal hemoglobinuria and other blood diseases. In Asia, membranous obstruction of the IVC is common, but the pathogenesis is unclear (18). Due to the obstruction of the proximal IVC, blood flow is slow, turbulent or reversed. This leads to the formation of a thrombus in these patients together with a hypercoagulable blood state (19).

BCS has been classified in a variety of ways. Wang *et al* classified BCS as three distinct types which have been accepted by the majority of the medical community: type A is primarily limited to stenosis or obstruction of the membrane, type B shows diffuse stenosis or obstruction of the IVC and type C shows hepatic vein occlusion. Interventional techniques have become the preferred method of treating BCS of type A and partial B and C types (1,20). There is no explicit classification to guide the clinical treatment of BCS with IVC thrombosis. The current classification is a modification of the classification by Wang *et al*. This classification is for BCS with IVC thrombosis.

On the basis of the Wang classification, 41 BCS patients with IVC thrombosis were divided into the three types according to the morphology of the thrombus, whether the thrombus was fresh or obsolete, and the relationship of the thrombus with the hepatic vein (Fig. 1). These may all be easily differentiated by color Doppler ultrasound and digital subtraction angiography of the IVC. On Doppler ultrasound scans, fresh thrombi are of low-echo density, whereas obsolete thrombi are of moderate to partially high-echo density (21).

The treatment principle of BCS is firstly the removal of the hepatic vein obstruction and the reduction of portal venous pressure, which is the key to preventing further development of cirrhosis and hepatic dysfunction, reducing or easing esophageal varices, avoiding rupture and bleeding of esophageal varices, liver and kidney failure later on and other problems (22). In accordance with this, various treatment methods were adopted based on the different types of patient and good treatment effects were achieved in this study. Twenty-eight patients (68.3%) were treated successfully with interventional therapy, 7 (17.1%) were given conservative treatment and 6 (14.6%) were treated with surgical shunts. Interventional therapy was used in 29 patients, all those of type I, type II and type III with acute thrombosis, and was successful in 28 (96.6%). None of these 28 patients had pulmonary embolism, pericardial tamponade or intra-abdominal bleeding. Four patients (9.8%) had a second dilation of IVC in the 1–5 year follow-up time.

The following precautions should be considered during the therapeutic procedure: i) Predilation with a balloon catheter of small caliber should be performed prior to the introduction of the thrombolytic catheter. For patients with BCS combined with IVC thrombosis, one-time angioplasty may cause the defluvium of large thromboses, leading to fatal pulmonary embolism. In the present cases, a guide wire and catheter were used to pass through the IVC occluded section and predilation was conducted with a balloon catheter of small caliber (5–7 mm diameter). This prevented pulmonary embolism caused by the defluvium of large thromboses, and facilitated the functioning of thrombolytic drugs by facilitating a through blood flow and preparing for the full dilation. This scheme is especially applicable for patients of type I. ii) For fresh and diffuse thrombosis, large mechanical catheter suction should be applied before the thrombolytic catheter is introduced. In patients of type II and III with IVC and diffuse thrombosis, mechanical large catheter suction is rapid and effective, and is able to remove most thrombi. A 12–14 F large catheter was used for such cases in our group. Direct manual lymphosuction and repeated operation is capable of removing most of the soft tissue of a fresh thrombus. The hemorrhage volume caused by catheter suction of the thrombus was <200 ml. There was no acute hemorrhage during and after surgery. The thrombus was sucked out into a thrombolytic catheter. Small doses of thrombolytic drugs (100,000 units of urokinase diluted in a 50-ml intravenous injection, 3 times a day) were then given. It is better to use pulse injection for thrombolytic drugs to increase the contact area between the drugs and the thrombus. Following thrombus suction and thrombolytic therapy, if there is residual mural thrombus, a stent should be used to apply mechanical compression and avoid its defluvium. iii) Avoiding the misdiagnosis of type II. For type II patients with IVC diffuse obsolete thrombosis, it is advisable to open the IVC at the end proximal to the hepatic vein opening as the operation is less complex. When performing a color ultrasound examination of BCS patients with IVC thrombosis, the surgeon and color ultrasound physicians should pay attention to the IVC near the opening of the hepatic vein to confirm whether it is type II and avoid a misdiagnosis of type III. iv) The problems related to an IVC stent. The clinical significance of maintaining hepatic vein patency is greater than that of keeping IVC patency. The hepatic vein occlusion caused by an IVC stent may make future possible interventions or radical surgery under direct vision more complicated. The selected stent should, therefore, be as short as possible for placing mechanical compression on the residual thrombus. When implanting an internal stent, any affect on the hepatic vein should be avoided. In patients with an unobstructed hepatic vein, where IVC occlusion or stenosis remains near the opening of the hepatic vein or there is residual stenosis even after balloon dilation, stent implantation should be avoided. If necessary, dilation may be performed again. v) Color ultrasound guidance is helpful for passing through the IVC. In cases where there is difficulty in passing through the IVC occlusion, color ultrasound guidance may be used together with fluoroscopy. Color ultrasound is able to show a full view of the pathologic changes and reveal the thrombi in the IVC and the hepatic vein, making pathologic changes in the route of the catheter visible. In addition, the IVC has a certain mobility during aspiration, and the stent implantation is performed more conveniently and accurately under the guidance of color ultrasound (23). As a result, in cases where pathological changes are visible they may be operated on and guided by color ultrasound. In more complex cases, it is better to combine the two together; this may increase the success rate and reduce intra-abdominal hemorrhage, pericardial tamponade and other complications. In addition, the form of the IVC occluded segment may also significantly guide the direction of rupture of membranes, which requires careful observation. The side with conical shape of the occluded segment is chosen to be punctured. When there are collateral vessels at the remote end of the occlusion and the puncture needle mistakenly enters collateral vessels during the rupture of membranes from bottom to top, the vessel may be ruptured during the balloon dilation, leading to abdominal hemorrhea. Consequently, proceeding from top to bottom is a more reliable method.

There are multiple limitations to this study due to its retrospective nature, however. The numbers are too small to draw a conclusion. In future a randomized trial will be performed in an attempt to verify the classification of the BCS patients with IVC thrombosis and the therapies indicated according to it.

This study indicates that the classification of BCS patients with IVC thrombosis is helpful in selecting the best therapeutic approach. Interventional therapy is the first therapeutic choice for BCS patients with IVC thrombosis of type I, type II and type III with acute thrombosis. In patients of type III with an obsolete thrombus, surgical shunts or conservative treatment are the main therapeutic methods.
